# Promising Anticancer Effect of Aurisin A Against the Human Lung Cancer A549 Cell Line

**DOI:** 10.31557/APJCP.2020.21.1.49

**Published:** 2020

**Authors:** Parichart Boueroy, Thidarut Boonmars, Somdej Kanokmedhakul, Sorujsiri Chareonsudjai, Ratsami Lekphrom, Suphachira Srichangwang

**Affiliations:** 1 *Faculty of Public Health, Kasetsart University Chalermphrakiat Sakon Nakhon Province Campus, Sakon Nakhon, *; 2 *Department of Parasitology, *; 3 *Cholangiocarcinoma Research Institute, Faculty of Medicine, *; 4 *Natural Products Research Unit, Department of Chemistry and Center of Excellence for Innovation in Chemistry, Faculty of Science, *; 5 *Department of Microbiology, Faculty of Medicine, *; 6 *Research Administration Division, Khon Kaen University, Khon Kaen, Thailand. *

**Keywords:** Lung anticancer, aurisin A, anti-migration, cell cycle arrest, apoptosis

## Abstract

**Objective:**

To investigate the anticancer effect of aurisin A and the underlying mechanisms of its action on the human lung cancer A549 cell line.

**Methods:**

Cell viability was determined by sulforhodamine B (SRB) assay, while cell cycle distribution and apoptosis were measured by ﬂow cytometry. The molecular underlying mechanisms of anti-cancer properties of aurisin A was determined by western blot analysis.

**Results:**

Aurisin A exerts its anticancer effects by inhibiting cell growth (p<0.001), increasing the proportion of cells at the G0/G1 phase (p<0.001), and decreasing the expression of cyclin D (p<0.05) and cyclin-dependent kinase 4 (Cdk-4) (p<0.001). Nuclear morphological changes were observed in aurisin A-treated cells, demonstrated by a dose-dependent increase in the number of apoptosis cells (p<0.001). After aurisin A treatment, B-cell lymphoma 2 (Bcl-2) was down-regulated (p<0.05), cleaved caspase-3 was up-regulated (p<0.05). In addition, aurisin A inhibits migration of cancer cells in a dose-dependent manner (p<0.001) and decreases the expression of epidermal growth factor receptor (EGFR) (p<0.05) and phosphorylated p38 (pp38) (p<0.05).

**Conclusion:**

These results indicated that in-vitro treatment of aurisin A against this human lung cancer cell line inhibits cell proliferation and migration, and induces apoptosis and cell-cycle arrest. Aurisin A is a promising anticancer agent for use against human lung cancer.

## Introduction

Lung cancer is one of the most common cancers in the world and an increasing cause of death in the United States of America (Brognard et al., 2001; Khuri and Cohen, 2004; Siegel et al., 2003; Govindan et al., 2012). Lung cancer has a high mortality rate because of its resistance to some current chemotherapeutic agents and difficulty of diagnosis (Brognard et al., 2001; Balsara et al., 2004; Hirsch et al., 2003). It is estimated about 1.8 million new cancer cases (13% of total cancer diagnoses) and more than 1 million related deaths per year (Torre et al., 2012). In the worldwide, lung cancer is the most common cause of death in men more than women (Pakzad et al., 2015). In addition, this cancer has a high incidence of recurrence. Non-small cell lung cancer (NSCLC) is the most common type of lung cancer, constituting approximately 75 - 85% of all cases. The usual chemotherapeutic agent for treatment of advanced NSCLC is the highly toxic platinum-based cisplatin. However, patients given this treatment have a low survival rate (Khuri et al., 2001; Li et al., 2004; Molina et al., 2006; Raez and Lilenbaum, 2004). Therefore, there is an urgent need for new therapeutic agents to treat lung cancer. The induction of apoptosis is a targeted strategy in effective drug design for cancer treatment, including human lung carcinoma (Singhal et al., 2005). Previously, aurisin A was shown to inhibit the growth of human small-cell lung cancer (NCI-H187) and cholangiocarcinoma (KKU-100, KKU-139, KKU-156, KKU-213, and KKU-214) cell lines. Moreover, aurisin A has various biological properties such as anti-malarial (*Plasmodium falciparum*) and anti-bacterial activities (*Mycobacterium tuberculosis*) (Kanokmedhakul et al., 2012). 

## Materials and Methods 

*Cell culture and plant material*

The human lung adenocarcinoma cell line (A549) (CCL-185), used as a model, was purchased from the American Type Culture Collection, MD, USA. Cancer cells were cultured in RPMI 1640 (Gibco; Thermo Fisher Scientific, Inc.) supplemented with 10% FBS, 100 units/ml of penicillin and 100 µg/ml streptomycin (Gibco BRL) at 37^o^C in 5% CO_2_ humidified atmosphere. Aurisin A has been extracted from culture medium of the luminescent mushroom *Neonothopanus nambi* PW1, and its molecular formula (C_30_H_36_O_9_) was deduced from HRESITOFMS (m/z 540.2257) (Kanokmedhakul et al., 2012). The culture liquid of PW1 (2.025 L) was extracted with EtOAc (2.5 L) yielded 2.1 g of yellow crystals of aurisin A. Yellow crystals of aurisin A were obtained after crystallization and slow evaporation from EtOAc follow as previous study (Kanokmedhakul et al., 2012). It was decomposed at 221.9^o^C and has a specific rotation, [α]_D_^26^ +701.3 (*c* 0.1, CHCl_3_). Aurisin A is soluble in dimethyl sulfoxide (DMSO) at 25 mg/mL and in 95% ethanol (EtOH) at 12.5 mg/mL. UV absorption at λ_max_ 331nm (logɛ 4.25) and 268 (logɛ 4.23) (Kanokmedhakul et al., 2012). Aurisin A was dissolved in DMSO to a concentration of 16 mM and further diluted to appropriate concentrations in the experiments. 5-FU (Boryung Pharmaceutic Co., Ltd. Korea) was aliquoted and kept at 4^o^C.

*Cell viability assay*

The effect of aurisin A on cell viability of human adenocarcinoma (A549) cell lines was determined using a sulforhodamine B (SRB) assay. Briefly, human adenocarcinoma (A549) cells were seeded into 96-well plates at 37^o^C. Cells were treated with aurisin A (0, 1, 2, 4, 8, 16, 32 µM) for 24, 48 or 72 h. After the treatment, cells were fixed, aspirated and incubated with SRB dye (Sigma Aldrich, Germany) for 30 min at room temperature. The protein-bound dye was solubilized by Tris base solution (10 mM, pH 10) (Sigma). The optical density (OD) was determined at 540 nm using a micro plate reader (ELISA Reader, Sunrise). The IC50 value was calculated from concentration-effect curves after linear regression analysis.

*Wound migration assay*

The human lung cancer A549 cells were seeded in 6-well plates and grown to 80 - 90% confluence. Monolayers of cells were wounded by scratching with a 1 mL plastic pipette and then rinsed with phosphate-buffered saline (PBS) to remove cell debris. Cells were incubated in medium containing 1% fetal bovine serum (FBS) with or without aurisin A (0, 6.25, 10.43 and 16.68 µM) for 24 or 48 h. The extent of the wound closure was monitored by microscopy and digitally photographed. The area of the wound was measured.

*Cell-cycle analysis*

*Human lung adenocarcinoma*

(A549) cells (1 × 10^5^ cells/well) were seeded in 6-well plates for 24 h and then treated with aurisin A (0, 6.25, 10.43 and 16.68 µM) and 5-FU (50 µg/ml). After 24 h, cells were harvested, washed, and fixed overnight in 70% ethanol at 4^o^C, and incubated at room temperature for 30 min in the dark with RNase A (final concentration 2 μg/mL) and propidium iodide (PI) (final concentration 2.4 μg/mL). The cell-cycle distribution was examined using a FACSCanto^TM^ II flow cytometer (BD Biosciences, San Jose, CA, USA) and the data were analyzed using FACSDiva^TM^ software (BD Biosciences). 

*Acridine orange/Ethidium bromide (AO/EB)*

Human lung adenocarcinoma (A549) cells were treated with various concentrations of aurisin A (0, 6.25, 10.43 and 16.68 µM) for 24 h. Cells were stained with 14 µl of 100 µg/ml of AO/EB mixture. Apoptotic cells with condensed or fragmented chromatin were observed with a confocal microscope. The percentage of apoptotic cells which are condensed chromatin or fragmented chromatin was calculated from 500 counted cells (Hahnvajanawong C et al., 2004).

*Apoptotic cell death detection assay via flow cytometry*

The effect of aurisin A on apoptosis induction in A549 cells was determined using an annexin-V-FLUOS staining kit (Roche Diagnostics, Penzberg, Germany). A549 cells (1 × 10^5^ cells/well) were treated with aurisin A (0, 6.25, 10.43 and 16.68 µM) and 5-FU (50 µg/ml) for 24 h. Floating and adherent cells were harvested by trypsinization, washed, and centrifuged to collect the supernatant. Cells were stained with annexin V-FITC (recombinant annexin V protein conjugated to green fluorescent FITC dye) and PI for 10 - 15 min at room temperature in the dark. The stained cells were then analyzed by flow cytometry.

*Protein extraction and western blot analysis*

The human lung adenocarcinoma (A549) cell line was cultured in 10 cm-diameter dishes (Costar^®^; Corning, Corning, NY, USA) and incubated with aurisin A (0, 6.25, 10.43, 16.68 µM) for 24 h. Harvested cells were lysed with ice-cold radioimmunoprecipitation assay (RIPA) buffer (50 mM Tris-HCl [pH 7.5], 150 mM NaCl, 0.5% Nonidet P-40 detergent, 1 mM EDTA, 1 mM dithiothreitol, 0.5% deoxycholate and 0.1% sodium dodecyl sulfate). The cell lysate was homogenized and clarified by centrifugation at 13,000 rpm for 30 min at 4^o^C. The protein concentration in the total cell lysate was determined by the Bradford method (Bradford, 1976). Cell lysates (5 µg of protein per lane) were fractionated by SDS-polyacrylamide gel electrophoresis (12% SDS-PAGE). The proteins were transferred onto a nitrocellulose membrane. The membrane was blocked and probed with primary antibody agents EGFR, pp38, Cyclin D, Cdk-4, Bax, Bcl-2, cleaved caspase-3 and β-actin, and then incubated with horseradish peroxidase-conjugated secondary antibody. The bound secondary antibodies on the nitrocellulose membrane were visualized using an enhanced chemiluminescence reagent (Pierce ECL; Thermo Fisher Scientific, Waltham, MA, USA), quantified by densitometry (ImageQuant LAS 4000; GE Healthcare, Chicago, IL, USA) and analyzed using the Scion Image program (Scion Corp., Frederick, MD, USA). The results were expressed as the relative density of protein normalized to β-actin.

*Statistical analysis*

Data are represented as mean ± SD of three independent experiments. Significant differences between the groups were analyzed by Student’s t-test using SPSS statistical software, version 16.0 (SPSS, Chicago, IL, USA). Statistical significance is indicated by *p<0.05, **p<0.01 or ***p<0.001.

## Results

*Aurisin A inhibits cancer cell proliferation in human lung cancer A549 cells*

**Figure 1:**
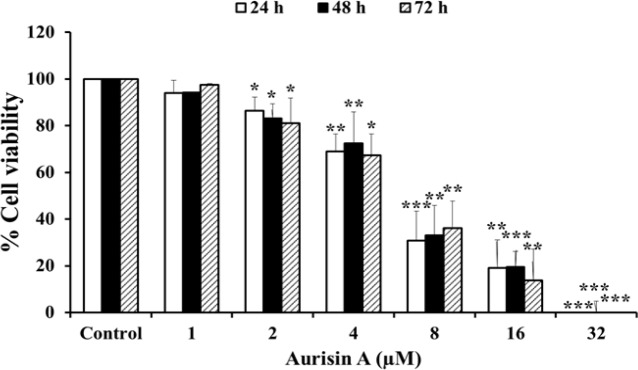
Growth Inhibitory Effect of Aurisin A on Human Lung Carcinoma (A549) Cells. A549 cells were treated with aurisin A (0, 1, 2, 4, 8, 16, 32 µM) on 24, 48, and 72 h. Viability of cells was determined by SRB assay. Each value represents the mean ± SD of three independent experiments. *p < 0.05, **p < 0.01, ***p < 0.001

The anticancer properties of aurisin A were determined by SRB assay. As shown in Figure 1, the human lung cancer cell line A549 was treated with aurisin A at various concentrations for 24, 48 or 72 h. Aurisin A was found to inhibit A549 viability in a time-dependent manner with IC50 10.43 ± 1.26, 10.60 ± 0.69, 9.2 ± 0.87, respectively.

*Aurisin A inhibited cancer cell migration in human lung cancer A549 cells*

**Figure 2:**
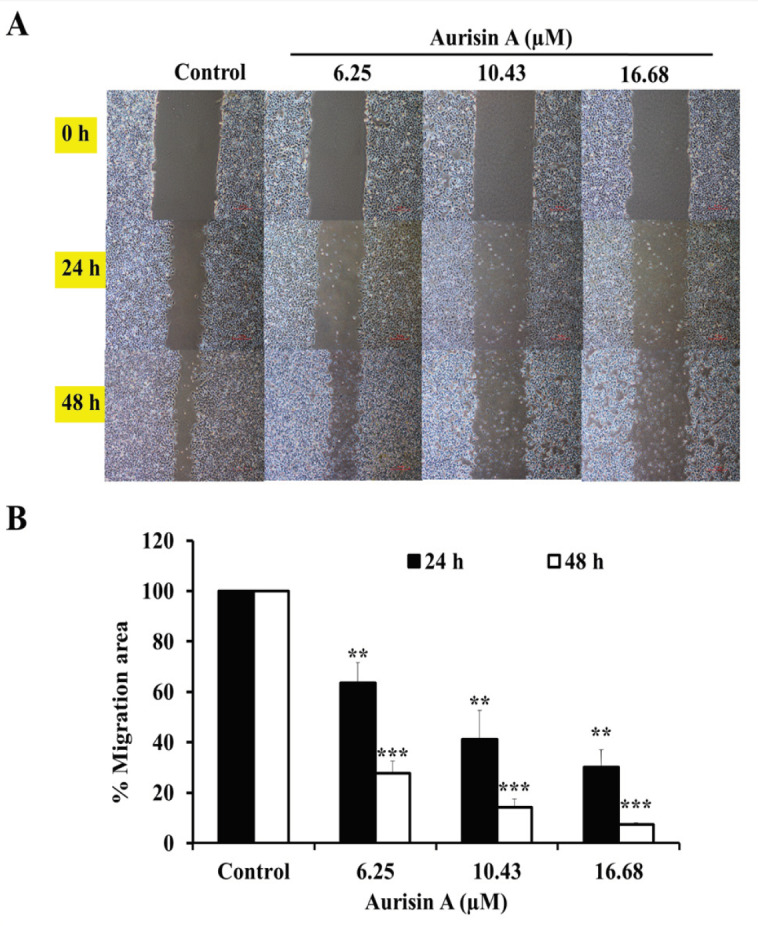
Anti-Migration Effect of Aurisin A on A549 Cells. Cells were seeded in 6-well plate. A Monolayer of cells were wounded and treated with aurisin A for 24 and 48 h. The wound area was determined by a phase-contrast microscope at 10×magnifications. The closure of gap distance was examined by (A) photographs and (B) calculated the percentage of migration area. **p < 0.01, ***p < 0.001

The effect of aurisin A on cell migration of A459 cells is shown in Figure 2. Aurisin A significantly inhibited cell migration into the wounded area in a dose- and time-dependent manner in all experimental dose. The percentage of migration area after treatment with aurisin A (16.68 µM) at 24 and 48 h was 30.25 ± 6.79% and 7.30 ± 0.72%, respectively, compared with the control group 100 ± 0.0% (Figure 2).

*Aurisin A-induced cell-cycle arrest in the G0/G1 phase*

**Figure 3:**
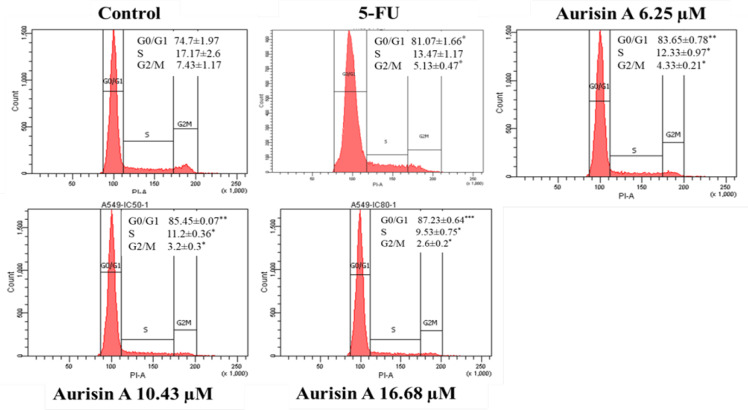
Cell Cycle Arrest in Aurisin A-Treated A549 Cells. A549 cells were treated with aurisin A and 5-FU for 24 h and stained nuclei with PI and analyzed by flow cytometry. The DNA content of cells was determined in three independent experiment. *p < 0.05, **p < 0.01, ***p < 0.001

The effect of aurisin A on cell-cycle distribution of the A549 cell line was determined by flow cytometry analysis. Aurisin A treatment significantly increased the population in the G0/G1 phase from 74.7 ± 1.97 % to 87.23 ± 0.64 % as compared to controls (Figure 3). The percentage of cells significantly decreased in the S phase (from 17.17 ± 2.6 % to 9.53 ± 0.75 %) and G2/M phase (from 7.43 ± 1.17 % to 2.6 ± 0.2 %), as shown in Figure 3. Fluorouracil (5-FU), the positive control, also significantly increased the proportion of cells in the G0/G1 phase from 74.7 ± 1.97 % to 81.07 ± 1.66 % as compared to negative controls (Figure 3).

*Aurisin A-induced apoptosis in human lung cancer A549 cells*

**Figure 4:**
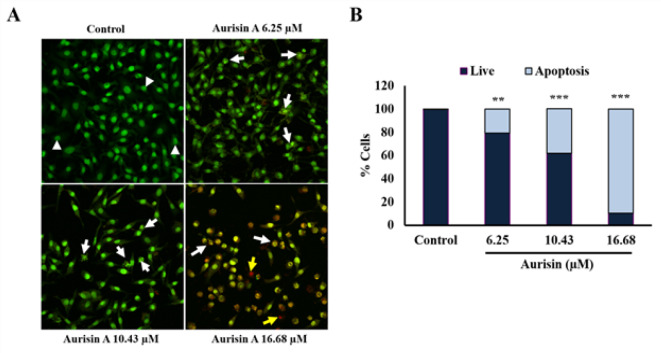
Nuclear Morphological Change of Aurisin A on A549 Cells. A549 cells were treated with aurisin A for 24 hr. (A) Cells were stained acridine orange/ethidium bromide and observed under a confocal microscope (magnification, x20). (B) Percentage of live and apoptotic cells in A549 cells treated with aurisin A. Normal cells were shown in arrow head, indicating normal with green homogeneous of the nucleus. Early apoptosis cells were shown in white arrow, indicating nuclear condensation and fragmentation. Late apoptosis cells were shown in yellow arrow, indicating cells that lose membrane integrity

**Figure 5:**
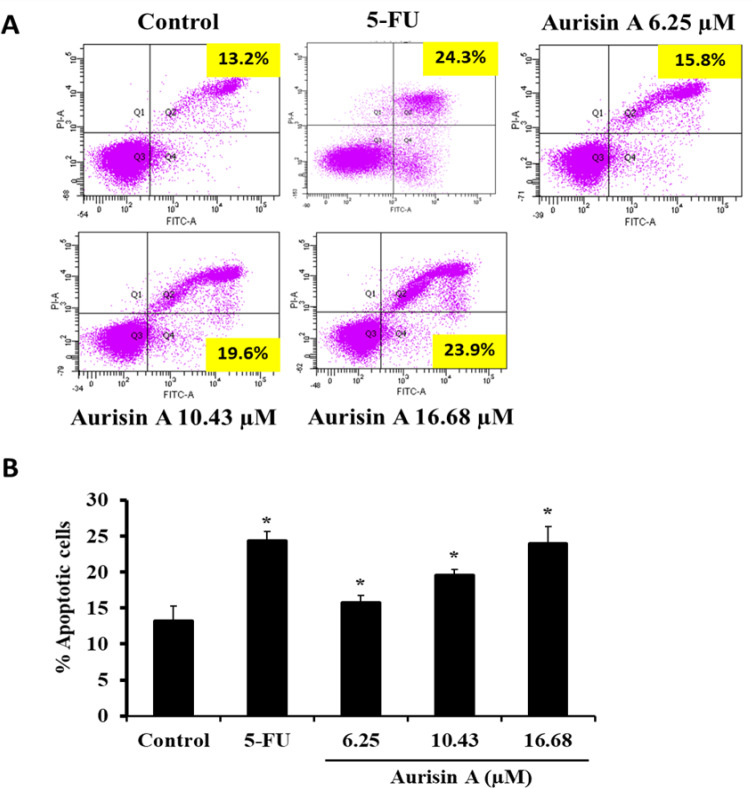
Apoptotic Effect of Aurisin A on A549 Cells. A549 cells were treated with aurisin A and 5-FU for 24 h. Cells were stained with Annexin V/PI and analyzed by flow cytometry. (B) The percentage of apoptotic cells was quantified by three independent experiment. *p < 0.05

The results of AO/EB staining were observed under a confocal microscope. Aurisin A-induced nuclear morphological changes included nuclear condensation and DNA fragmentation, as shown by white arrowheads in Figure 4A. Late apoptotic or necrotic cells were observed in the aurisin A-treated group, as shown stained red in Figure 4A. The percentage of apoptotic cells was significantly increased in aurisin A treated cells compared to untreated cells (Figure 4B). Consistent with flow cytometry analysis in annexin V/PI staining, treatment with aurisin A significantly increased the percentage of apoptotic cells from 13.2 ± 2.07% to 23.93 ± 2.41% as compared to controls (Figure 5). Also, treatment with 5-FU significantly increased the proportion of apoptotic cells (from 13.2 ± 2.07 to 24.3 ± 1.32) as shown in Figure 5.

*Effect of aurisin A on the expression of proteins associated with cell migration, cell cycle and apoptosis*

**Figure 6.**
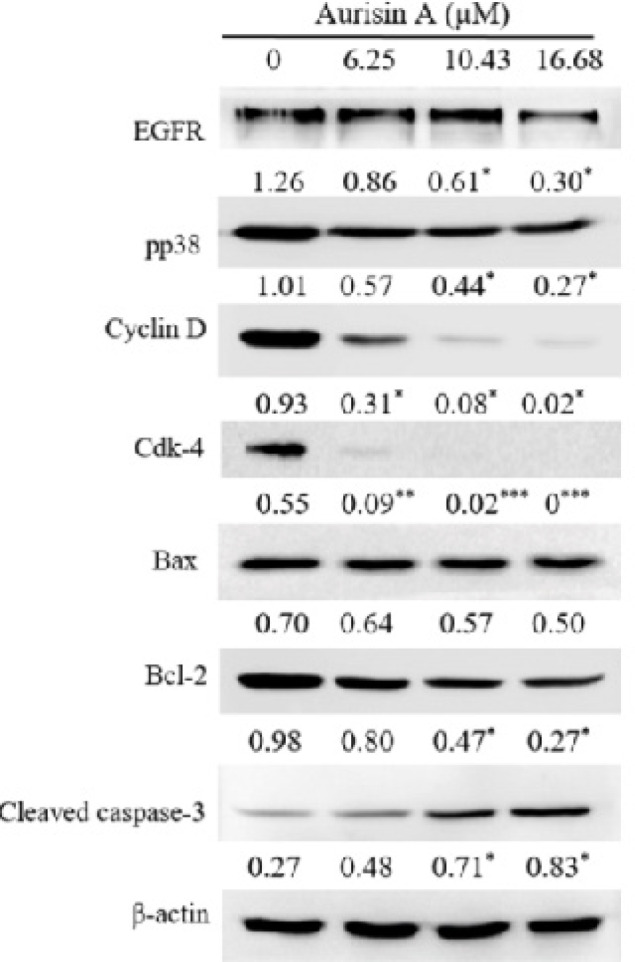
Changed the Protein Expression Related to Cell Migration, G0/G1 Arrested, and Apoptosis in A549 Cells. A549 cells were treated with aurisin A for 24 h. The expression of cell migration-related molecules, EGFR and pp38, cell cycle arrest-related molecules, Cyclin D and Cdk-4 and apoptosis-related molecules Bax, Bcl-2 and Cleaved caspase-3 were normalized relative to β-actin. Data are presented as mean ± SD of three separate experiments. *p < 0.05, **p < 0.01, ***p < 0.001

As shown in Figure 6, the expression of proteins regulating cell migration (epidermal growth factor receptor (EGFR) and phosphorylated p38) was downregulated after treatment with aurisin A. In keeping with the ability of aurisin A to induce cell-cycle arrest at the G0/G1 phase, the expression of cell cycle-regulated proteins cyclin D and Cdk-4 was significantly decreased as compared to controls (Figure 6). Similarly, the expression of anti-apoptotic protein Bcl-2 was significantly decreased and cleaved caspase-3 was up-regulated, but the pro-apoptotic protein Bax was not affected (Figure 6).

## Discussion

This present study shows that aurisin A has promise as an anti-cancer agent, as evidenced by; i) inhibited cell proliferation and cancer cell migration (decreased EGFR and pp38 expression), ii) induced apoptosis (up-regulated caspase-3) and iii) cell cycle arrest at the G0/G1 phase with decreased expression of the proteins cyclin D and Cdk-4.

Previous studies reported about natural active compound can induce apoptosis and cell cycle arrest in human lung cancer (Peng et al., 2016; Su et al., 2014). Dysregulation of cell proliferation, together with inhibition of the apoptosis pathway, is associated with tumorigenesis (Evan and Vousden, 2001). Induction of apoptosis in the tumor is a desired result of treatment with chemotherapeutic agents and radiation. The apoptosis pathway has several cellular and molecular biological features including cell shrinkage, nuclear and chromatin condensation, externalization of phosphatidyl serine (PS) and activation of caspase (Martin et al., 1995; Salvesen and Dixit, 1997; Goldar et al., 2015). We determined morphological changes by observation of cells stained with AO/EB, which allowed us to see condensation and fragmentation of DNA in A549 cells after treatment with aurisin A. Central regulators in the apoptotic family either inhibit (Bcl 2 and Bcl extra large) or promote (Bax and Bak) programmed cell death (Daniel and Korsmeyer, 2004; Liang et al., 2011; Hata et al., 2015). The balance between the levels of pro- and anti-apoptotic proteins is important for cell survival or programmed cell death (Peng et al., 2016). In this study, we found that aurisin A-mediated induction of apoptosis in A549 cells was associated with decreased expression of Bcl-2.

Activation of caspases is important for induction of apoptosis (Nicholson and Thornberry, 1997). The caspase family is divided into initiator and activator caspases. Initiator caspases, such as caspase-1, -2, -8, -9, and -10, initiate a proteolytic cascade while activated caspases, such as caspase-3, -6, and -7, are associated with cleavage of specific intracellular substrates (focal adhesion kinase, poly-ADP-ribose polymerase) (Salvesen and Dixit, 1997; Nicholson and Thornberry, 1997; Alnemri et al., 1996; Hu et al., 2013). We found that aurisin A can induce the externalization of PS and activate cleaved caspase-3. Caspase-3 is essential for DNA fragmentation leading to morphological changes associated with apoptosis (Janicke et al., 1998), changes which were evident in A549 cells treated with aurisin A. 

Inhibition of cancer cell migration is one of the therapeutic targets of effective anti-cancer drugs. Mitogen-activated protein kinases (MAPK) fall into three subfamilies; extracellular signal-regulated kinases (ERK), Jun NH_2_-terminal kinases (JNK), and p38 kinases. These pathways are important in tumor progression and metastasis (Reddy et al., 2003). The EGFR signal-transduction pathway is an important target for anticancer drugs. EGFR is a receptor of tyrosine kinase that is overexpressed in various types of cancer such, as breast, head and neck, ovarian and non-small cell lung cancer (Salomon et al., 1996; Huang and Fu, 2015). Recognition of EGFR plays an important role in inflammation, cell survival, cell adhesion, cell motility, invasion and angiogenesis (Woodburn, 1999). Verbeek et al., (1998) reported that overexpression of EGFR can induce cell migration in human breast cancer. We found that aurisin A can inhibit the migration of A549 cells by decreasing the expression of migration regulating protein pp38 and EGFR. 

In conclusions, we conclude that aurisin A exerts cytotoxic effects against human lung carcinoma. This effect is associated with the inhibition of cancer cell migration, and induction of cell-cycle arrest and apoptosis in A549 cells. At the molecular level, aurisin A causes changes in the expression of migration-related proteins EGFR and pp38, G0/G1 phrase-related proteins cyclin D and Cdk-4, and apoptosis-regulated proteins Bcl-2 and cleaved caspase-3. These results indicate that aurisin A has potential as a drug treatment for human lung carcinoma.

## Conﬂict of interests

No competing of interests exist.
